# Nerve abnormalities in lumbar disc herniation: A systematic review and meta-analysis of diffusion tensor imaging

**DOI:** 10.1371/journal.pone.0279499

**Published:** 2022-12-27

**Authors:** Nan Wang, Daoxi Sun, Xiaoyu Zhang, Zhipeng Xi, Jingchi Li, Lin Xie

**Affiliations:** Department of Spine Surgery, Affiliated Hospital of Integrated Traditional Chinese and Western Medicine for Nanjing University of Chinese Medicine, Nanjing, Jiangsu Province, P.R. China; Lewisham University Hospital, UNITED KINGDOM

## Abstract

**Purpose:**

The purpose of this study was to examine the values of fractional anisotropy (FA) and apparent diffusion coefficient (ADC) in diffusion tensor imaging (DTI) for diagnosing patients with nerve impairment due to lumbar disc herniation (LDH).

**Methods:**

A literature search of databases (PubMed, Web of Science, Cochrane Library and Embase) was systematically performed to identify articles published before September 2021 that were relevant to this study. FA and ADC estimates of compressed nerve roots due to LDH and healthy controls in the same segment were compared, with either fixed or random effects models selected according to I^2^ heterogeneity. Additionally, subgroup analysis, sensitivity analysis, potential publication bias analysis and meta-regression analysis were also performed.

**Results:**

A total of 369 patients with LDH from 11 publications were included in this meta-analysis. The results showed significantly lower FA values (Weighted Mean Difference (WMD): -0.08, 95% confidence interval (CI): -0.09 to -0.07, P ≤ 0.001, I^2^ = 87.6%) and significantly higher ADC values (WMD: 0.25, 95% CI: 0.20 to 0.30, P ≤ 0.001, I^2^ = 71.4%) of the nerve on the compressed side due to LDH compared to the healthy side. Subgroup analysis indicated that different countries and magnetic field strengths may be associated with higher heterogeneity. Furthermore, meta-regression analysis further revealed that segment and field strength did not have a significant effect on the results, regardless of the FA or ADC values. Contrastingly, in FA, the year of publication, country, b value and directions showed an effect on the results.

**Conclusions:**

This meta-analysis showed a significant decrease in FA and a significant increase in ADC in patients with nerve damage due to LDH. The results favourably support the presence of nerve impairment in patients with LDH.

## Introduction

Lumbar disc herniation (LDH) is clinically characterized by sciatica and low back pain (LBP), which severely affect the quality of life and impose a serious burden on the healthcare system and patients’ families [1,2]. Sciatica is caused by the compression of the sciatic nerve with a herniated intervertebral disc. Additionally, epidemiological investigations have reported that LDH is the most common cause of nerve root compression in the lumbosacral region [3]. The main signs and symptoms of LDH include rhizomatic pain, hyperalgesia and incompetence in the circulation of a nerve root in one or more lumbosacral regions [4]. Magnetic resonance imaging (MRI) is the standard indicator for evaluating LDH, with high accuracy and reliability rates [5]. Although conventional MRI is widely used clinically, it is difficult to assess the severity of nerve root compression based solely on an MRI [6]. Clinicians may observe symptoms that are significantly inconsistent with the imaging findings. Patients without significant nerve root compression on imaging may have severe clinical signs of nerve root compression whereas those with severe compression may not have significant clinical signs. Additionally, patients with untreated herniated lumbar discs may also exhibit complex clinical symptoms. Therefore, it can be hypothesised that the mechanical compression of the lumbar disc on MRI is not the only cause of low back pain and sciatica in patients.

Diffusion tensor imaging (DTI) is a proven functional magnetic resonance imaging (fMRI) technique, which reflects the internal microstructural characteristics of tissues more accurately than other MRIs by calculating the anisotropy of water molecule diffusion in tissues. This technique is currently widely used in various fields; however, it is mainly used in the evaluation of central nervous system lesions [7], peripheral nervous system lesions [8], spinal cord lesions [9], intracranial vascular diseases [10] and other nerve-related diseases. Apparent dispersion coefficient (ADC) and fractional anisotropy (FA) are two important parameters of DTI in the observation of nerve lesions. FA measures the directionality of spreading and details the integrity of the fibres while ADC reflects the diffusivity of molecules in 3D tissue space [11]. Moreover, the degree of nerve root compression has been correlated with FA and ADC values [3]. The higher the degree of nerve root compression, the lower the FA value and the higher the ADC value. Therefore, FA and ADC values can reflect the pathophysiological state of nerve fibre bundles caused by LDH [12].

Previous studies have reported encouraging results regarding the use of DTI in the diagnosis of nerve injury due to LDH; however, variations exist in DTI values across segments and with different degrees of compression. Additionally, various factors such as magnetic field strength, measurement technique, scanning parameters and position choice can vary the sensitivity of the DTI results [13–15]. Several studies have also reported that FA values are generally decreased after a herniated disc compresses a nerve [14,16,17]. However, it remains controversial whether ADC values increase after a nerve injury [18,19]. Consequently, this review demonstrates the importance of ADC and FA values in DTI in the diagnosis of nerve compression due to LDH. This systematic meta-analysis is divided into two main aspects. The first aspect clarifies the changes in the DTI parameters (mainly FA and ADC values) of the nerve on the compressed side compared to the healthy side. The second aspect uses meta-regression to explore the effects of publication year, study country nerve root compression segment and scanning parameter settings on DTI outcomes.

## Materials and methods

### Literature search and data sources

This study was designed and reported following the PRISMA Declaration Guidelines [20]. Studies in the PubMed, Web of Science, Cochrane library and Embase database were searched using the following search terms: ‘Diffusion tensor imaging’, ‘DTI’, ‘Imaging, Diffusion Tensor’, ‘Diffusion Tensor MRI’, ‘Diffusion Tensor MRIs’, ‘MRI, Diffusion Tensor,’ ‘DTI MRI’, ‘Diffusion Tractography’, ‘Tractography, Diffusion’, ‘lumbar disc herniation’, ‘LDH’, ‘lumbar disc protrusion’, ‘lumbar disk herniation’, ‘lumbar herniated disk’, ‘lumbar intervertebral disc herniation’, ‘lumbar intervertebral disc prolapse’ and ‘prolapse of lumbar intervertebral disc.’ Articles published before September 2021 were retrieved. Furthermore, two reviewers independently screened the titles and abstracts of the research studies to ensure precise results. The search results were imported to EndNote X9 software for further treatment (Table 1).

**Table 1:** Search strategy in the Pubmed database.

Number	Search contents
#1	Diffusion tensor imaging[MESH]
#2	(Imaging, Diffusion Tensor) OR (Diffusion Tensor Magnetic Resonance Imaging) OR (Diffusion Tensor MRI) OR (Diffusion Tensor MRIs) OR (MRI, Diffusion Tensor) OR (DTI MRI) OR (Diffusion Tractography) OR (Tractography, Diffusion)
#3	(lumbar disc herniation) OR (LDH) OR (lumbar disc protrusion) OR (lumbar disk herniation) OR (lumbar herniated disk) OR (lumbar intervertebral disc herniation) OR (lumbar intervertebral disc prolapse) OR (prolapse of lumbar intervertebral disc)
#4	(#1 OR #2) AND #3

### Inclusion and exclusion criteria

Inclusion criteria were as follows: (1) studies reporting DTI indexes in patients with LDH who have FA or ADC values on the affected side versus healthy controls; (2) diagnosis of LDH with nerve compression based on patient symptoms, signs and imaging; (3) original clinical articles. Exclusion criteria were as follows: (1) unclear expression of the measurement methods of FA and ADC values; (2) incomplete information; (3) missing data; (4) case reports, duplicate literature and studies using animals as study subjects.

### Data extraction and quality assessment

Two authors independently performed a quality assessment of the included literature, extracting essential information and data for analysis. Disagreements were resolved by consensus after discussion, and if still controversial, a third reviewer was introduced to join the discussion. Demographic characteristics (age, sample size, country, gender), clinical data (prominent segments), indicators of measurement and imaging parameters were extracted (Tables 1 and 2), and the quality of the included literature was evaluated according to Newcastle–Ottawa Scale (NOS) [21].

**Table 2:** Demographic and clinical characteristics of the participants in the 11 studies.

Study	Country	Design	Age(Year)	Age range	Gender(M,F)	Patients	compressed nerve root	DTI parameters
Zeng 2018 [22]	China	CCS	38.2	18–61	38/26	64	L3,L4,L5	FA
Chen 2020 [23]	China	CCS	40.23	20–60	25/15	40	S1	FA、ADC
Cui 2017 [24]	China	CCS	43	20–60	14/11	25	L5,S1	FA、ADC
Li 2014 [16]	China	CCS	44.7	21–63	11/9	20	L4,L5,S1	FA、ADC
Zhang 2018 [14]	China	CCS	27.7±5.5	18–42	25/15	40	L5,S1	FA、ADC
Shi 2020 [17]	China	CCS	59.1±13.3	29–85	21/15	36	L5,S1	FA
He 2018 [25]	China	CCS	38	22–58	11/9	20	L4,L5,S1	FA、ADC
Shi 2021 [26]	China	CCS	42.1±15.4	NA	36/30	66	L4,L5,S1	FA、ADC
Balbi 2011 [27]	France	CCS	44.7	21–63	9/10	20	L5,S1	FA、ADC
Li 2019 [28]	China	CCS	35	26–53	12/7	19	L5,S1	FA
Liu 2020 [29]	China	CCS	34	26–53	12/7	19	L5,S1	FA、ADC

Note: M: Male; F: Female; CCS: Case control study; DTI: Difusion tensor imaging; FA: Fractional anisotropy; ADC: Apparent difusion coefcient; NA: Not available; L: Lumbar; S: Sacral.

### Statistical analysis

Stata 14 software was used for quantitative merging. Weighted Mean Difference (WMD) values and 95% confidence interval (CI) were used as the combined effect indicators, and the chi-square test combined with the I^2^ indicator was used to test the heterogeneity of the included studies. P < 0.1 and I^2^ > 50% indicated heterogeneity between studies, and the random-effects model was used; however, P ≥ 0.1 and I^2^ ≤ 50% indicated no significant heterogeneity between studies, and the fixed effect model was used. Sensitivity and subgroup analyses were used to find the possible sources of heterogeneity. Publication bias was assessed by observing the symmetry of the funnel plot and Egger’s graph. The effect of multiple variables on outcome was evaluated using meta-regression analysis. Additionally, inter-rater reliability and Cohen’s kappa were used for quality assessment.

## Results

### Included studies

**Fig 1:**
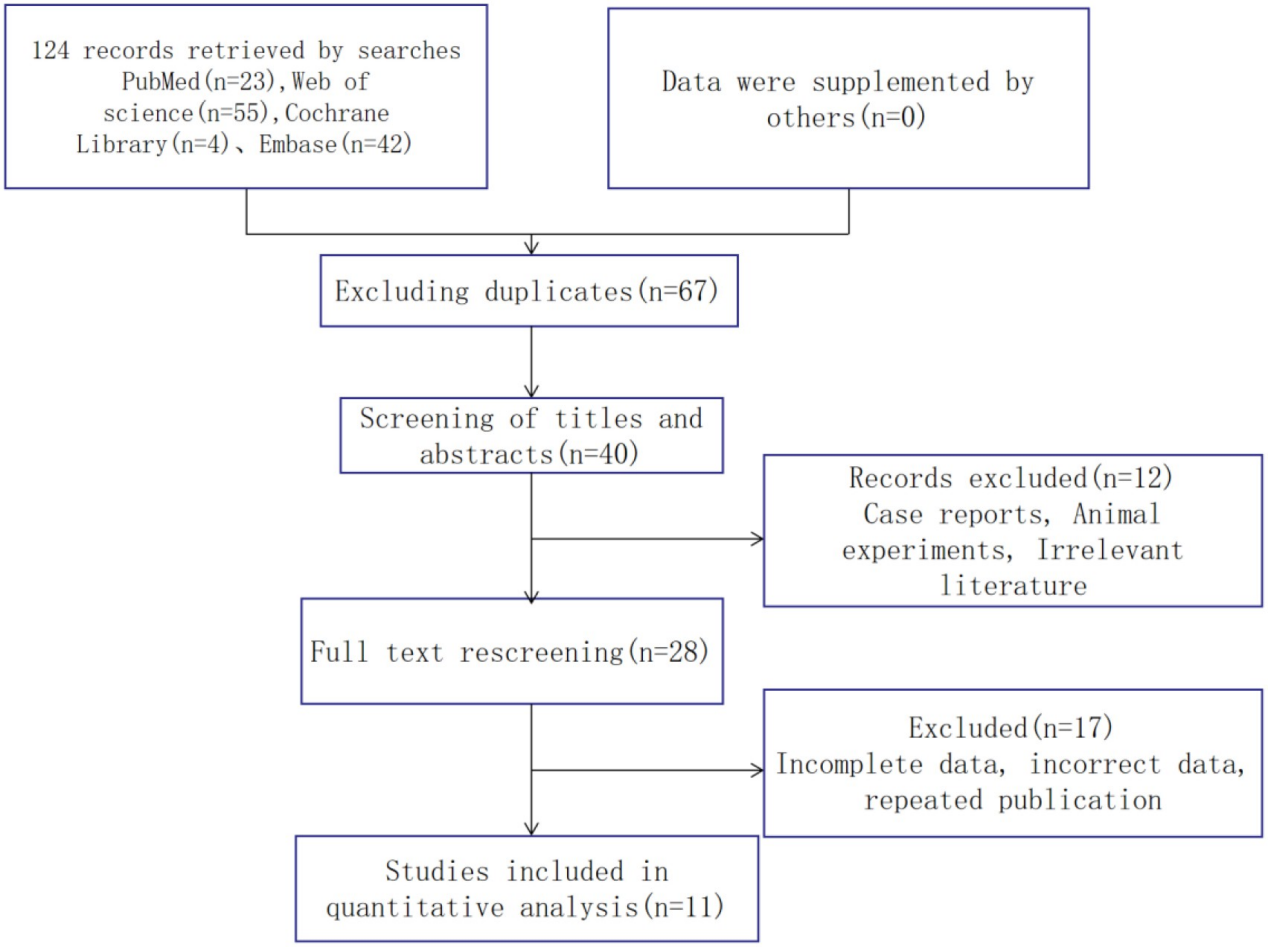
Flow chart of the study process.

A total of 11 English-language publications, including 369 patients with LDH, were included in this meta-analysis, and the literature search process and results are shown in Fig 1. FA values were reported in all 11 studies included in the meta-analysis, but ADC values were reported in only eight studies. The characteristics of all general studies and imaging parameters included in the literature are shown in Tables 2 and 3, respectively. The results of the literature quality assessment using the NOS tool are shown in Table 4.

**Table 3:** The characteristics of imaging parameters in the 11 studies.

Study	Field strength	Directions(number)	b value(s/mm2)	TE(ms)	TR(ms)	FOV(mm*mm)	Matrix(mm)	slice thickness(mm)	slice gap(mm)	scan time
Zeng 2018 [22]	3.0T	11	800	76	6000	320*256	NA	3	0	4min54s
Chen 2020 [23]	1.5T	15	600	90	1950	250*346	84*116	2.5	0	7min25S
Cui 2017 [24]	3.0T	NA	600	95	7500	230*230	NA	3	0	NA
Li 2014 [16]	1.5T	32	800	90	4900	224*224	76*89	2	0	10min
Zhang 2018 [14]	3.0T	20	700	88	11600	240*100	128*128	1	1	8min35s
Shi 2020 [17]	3.0T	11	800	80	6000	420*420	96 *128	4	0	7min24s
He 2018 [25]	3.0T	32	800	90	4900	224*224	76*88	2	0.8	10min
Shi 2021 [26]	3.0T	11	800	80	6000	420*420	96*128	4	0	7min24s
Balbi 2011 [27]	1.5T	25	900	94	3433	200*200	112*111	3	0	12min06S
Li 2019 [28]	3.0T	12	800	95	8000	350*350	NA	4	0.78	NA
Liu 2020 [29]	3.0T	12	800	95	8000	350*350	NA	4	NA	NA

Note: T: Tesla; NA: Not available; FOV: Field of voxel; TR: Repetition time; TE: Echo time; mm: Millimeter; min: Minute; s: Second.

**Table 4:** NOS evaluation score table in the 11 studies.

Study	Year	S				C		E			Total score
		S1	S2	S3	S4	C1	C2	E1	E2	E3	
Zeng	2018	1	1	0	1	1	1	1	1	1	8
Chen	2020	1	0	0	1	1	1	1	1	1	7
Cui	2017	1	1	0	1	1	1	1	1	1	8
Li	2014	1	1	0	1	1	1	1	1	1	8
Zhang	2018	1	1	0	1	1	1	1	1	1	8
Shi	2020	1	1	0	1	1	1	1	1	1	8
He	2018	1	1	0	1	1	1	1	1	1	8
Shi	2020	1	1	0	1	1	1	1	1	1	8
Balbi	2011	1	1	0	1	1	0	1	1	1	7
Li	2019	1	1	0	1	1	1	1	1	1	8
Liu	2020	1	1	0	1	1	1	1	1	1	8

Note: S: Case selection, S1: Case determination, S2: Case typicality, S3: Control selection, S4: Control determination; C: Baseline comparability, C1: Control for significant confounders, C2: Control for any confounders; E: Exposure factors, E1: Exposure determination, E2: Case, control exposure determination identical, E3: Non-response rate, NOS: Newcastle–Ottawa Scale.

### Meta-analysis of LDH induced DTI changes(FA and ADC values)

**Fig 2:**
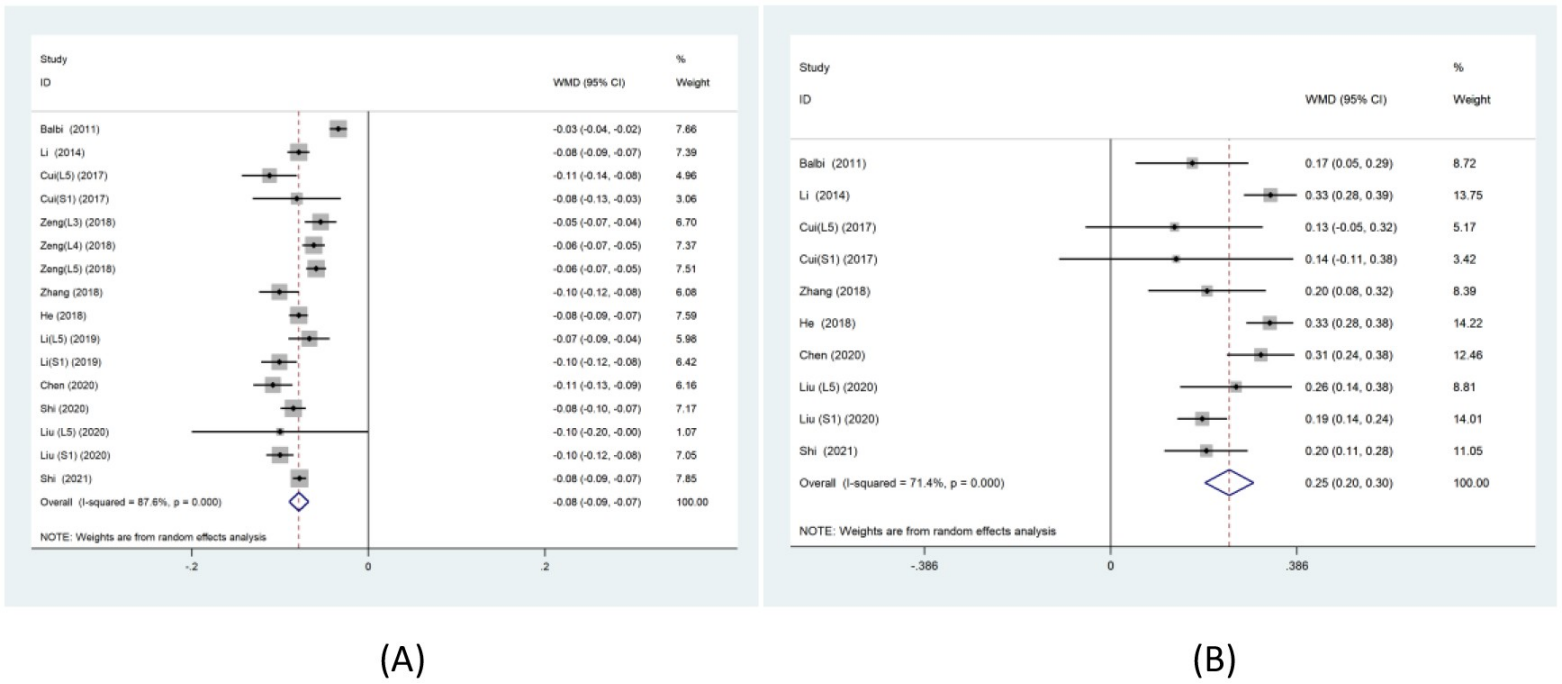
The forest plot shows the comparison of FA values(A) and ADC values(B) between the stressed and healthy sides in the random effects model.

A meta-analysis of FA values in the included studies revealed that the nerve compression side of patients with LDH had significantly lower FA values compared to the healthy side (WMD: -0.08, 95% CI: -0.09 to -0.07, P ≤ 0.001, I^2^ = 87.6%; Fig 2A). However, the eight publications reporting ADC values showed significantly higher values on the affected side of patients with LDH compared to the healthy side (WMD: 0.25, 95% CI: 0.20 to 0.30, P ≤ 0.001, I^2^ = 71.4%; Fig 2B). Moreover, owing to the high heterogeneity of the model, a random effects model was used for subsequent analysis.

### Subgroup analyses

**Fig 3:**
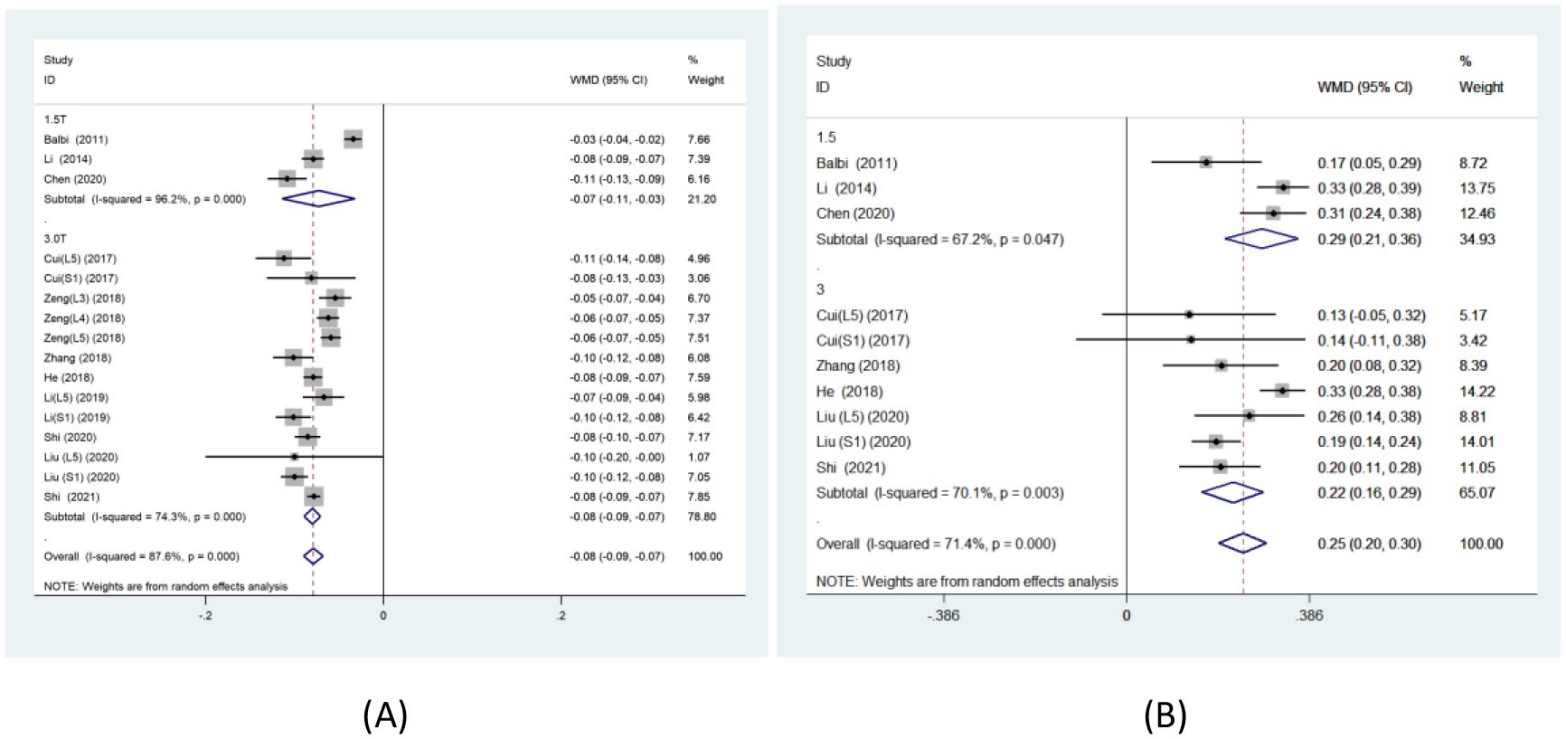
The forest plot shows subgroup of field strength about FA values(A) and ADC values(B).

Owing to the high heterogeneity in this study, the origin of heterogeneity was evaluated using subgroup analysis, focusing primarily on magnetic field strength. Subgroup analysis revealed that patients with LDH had significantly lower FA values on the affected side than on the healthy side(1.5T: WMD: -0.07, 95% CI: -0.11 to -0.03, P ≤ 0.001, I^2^ = 96.2%; 3.0T: WMD: -0.08, 95% CI: -0.09 to -0.07, P ≤ 0.001, I^2^ = 74.3%)(Fig 3A). However, based on field strength, subgroup analyses revealed that patients with LDH had significantly higher ADC values on the affected side than on the healthy side (1.5T: WMD: 0.29, 95% CI: 0.21 to 0.36, P = 0.047, I^2^ = 67.2%; 3.0T: WMD: 0.22, 95% CI: 0.16 to 0.29, P = 0.003, I^2^ = 70.1%) (Fig 3B).

These results suggest that magnetic field strength, although it has some influence on heterogeneity, is not the primary reason for the higher heterogeneity in this study. Moreover, the results of the subgroup analysis are consistent with that of the meta-analysis.

### Publication bias

**Fig 4:**
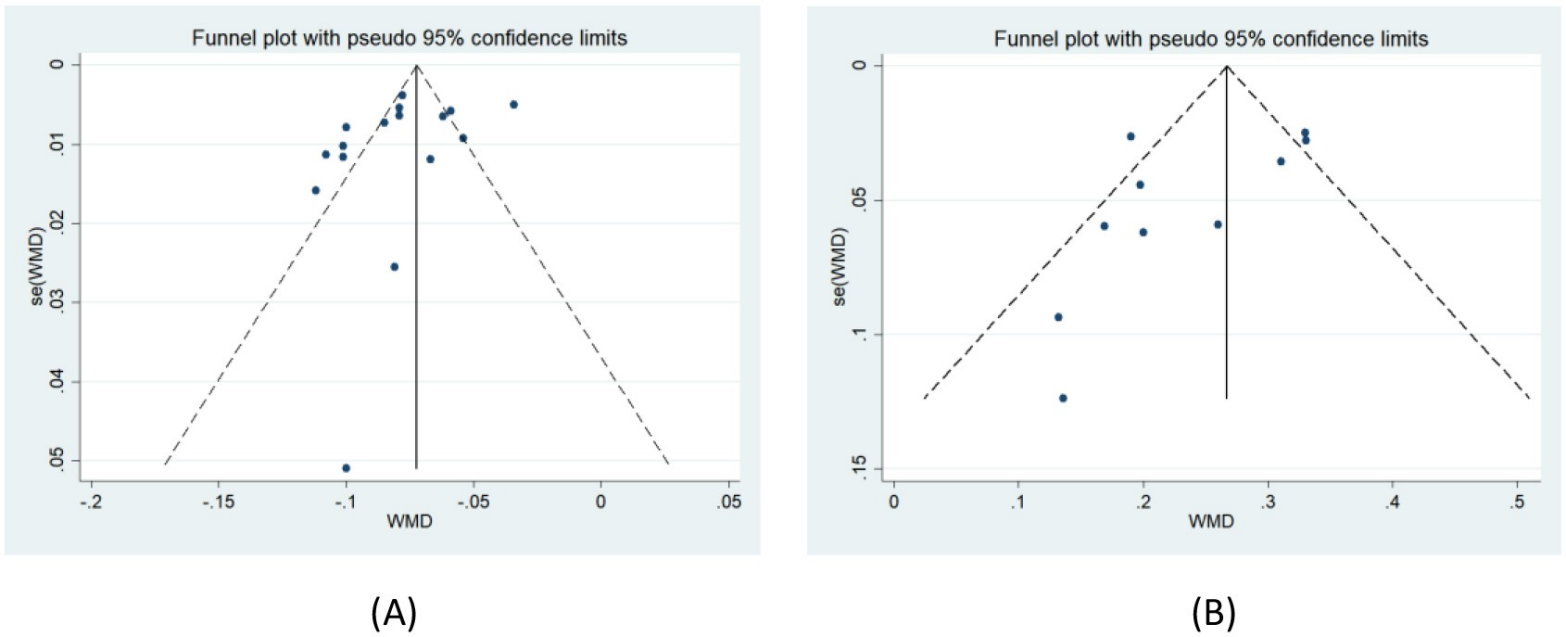
Funnel plot of FA(A) and ADC(B) values between the compressed and healthy sides of LDH patients.

**Fig 5:**
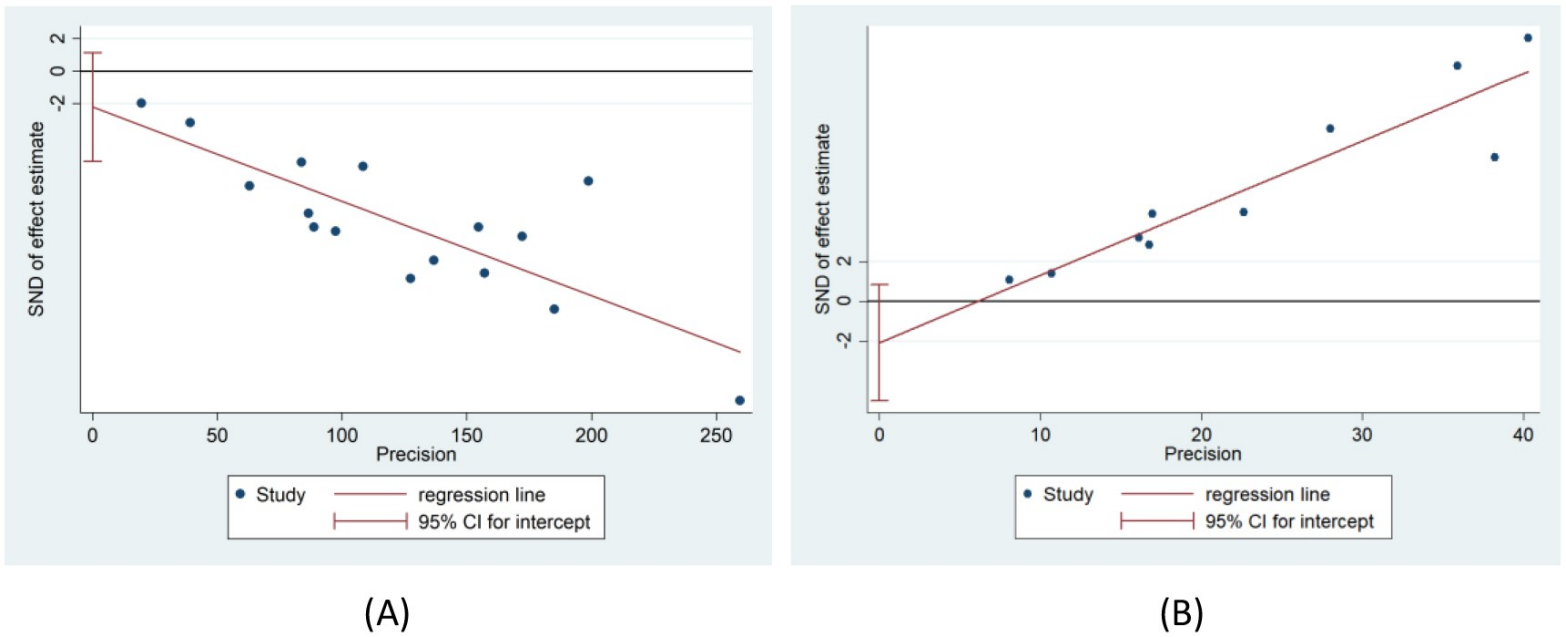
Egger graph of FA(A) and ADC(B) values between the compressed and healthy sides of LDH patients.

The funnel plots for FA (Fig 4A) and ADC (Fig 4B) show a possible risk of publication bias in this study. As can be seen in the figures, although the funnel plot for FA is slightly symmetrical, there is missing data in the lower half of the overall, as well as in the lower right corner of ADC. To test the asymmetry of the funnel plot, we also performed an Egger’s test. Furthermore, Egger’s graph shows that the FA (Fig 5A) (P = 0.178 > 0.05) and ADC (Fig 5B) (P = 0.140 > 0.05) values are almost symmetrical on both sides of the regression line, indicating that the possibility of publication bias is low.

### Sensitivity analysis

**Fig 6:**
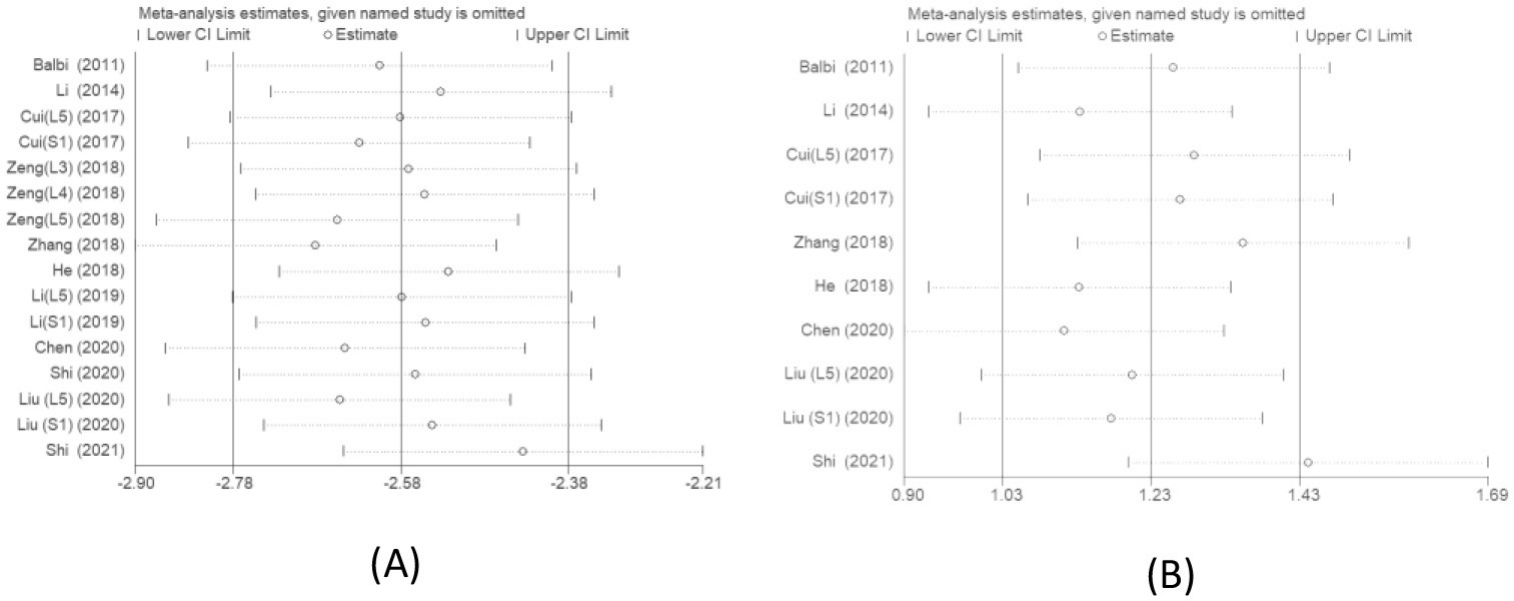
Sensitivity analysis graph of FA(A) and ADC(B) values between the compressed and healthy sides of LDH patients.

Sensitivity analysis revealed that both FA (Fig 6A) and ADC (Fig 6B) showed good robustness in this study.

### Meta-regression analysis

Data from the included publications were used for meta-regression analysis. The variables including the year of publication, country, compression segment, b vale, field strength and directions in the study were used to clarify whether these factors affected the results of this review. Meta-regression analysis of FA values demonstrated that the P values of the publication year, country, b value and directions were less than 0.05 (Table 5), indicating the effect of these variables to a certain extent on the results of ADC. Furthermore, we also performed a meta-regression analysis of these six factors in the study of ADC values. The results of the meta-regression of ADC values showed that the p-values were greater than 0.05 for all included factors (Table 6), indicating that there was no significant effect of these factors on the results of ADC.

**Table 5:** Meta-regression analysis of FA values in DTI.

_ES	Coef.	Std. Err.	t	P>|t|	95% Conf. Interval	
Year of publication	-.0045856	.0018494	-2.48	**0.026**	-.0085522	-.0006191
_cons	9.17497	3.731826	2.46	0.028	1.171	17.17894
Country	-.0478826	.0170181	-2.81	**0.014**	-.0843829	-.0113824
_cons	.0138826	.0329834	0.42	0.680	-.0568596	.0846249
Segment	-.0004493	.0040249	-0.11	0.913	-.009082	.0081834
_cons	-.077549	.0132616	-5.85	0.000	-.1059923	-.0491056
B value	.0001998	.0000553	3.61	**0.003**	.0000812	.0003183
_cons	-.2332299	.0432768	-5.39	0.000	-.3260493	-.1404104
Field strength	-.0094671	.0143689	-0.66	0.521	-.0402852	.0213511
_cons	-.0618766	.0263639	-2.35	0.034	-.1184216	-.0053316
Directions	-.0073108	.0028656	-2.55	**0.023**	-.013457	-.0011647
_cons	-.0487873	.0124589	-3.92	0.002	-.075509	-.0220656

Note: Coef: Coefficient; Std. Err: Standard error; Conf. Interval: Confidence interval; _cons: Constant.

**Table 6:** Meta-regression analysis of ADC values in DTI.

_ES	Coef.	Std. Err.	t	P>|t|	95% Conf. Interval	
Year of publication	-.0002122	.0088169	-0.02	0.981	-.0205439	.0201195
_cons	.6732106	17.79055	0.04	0.971	-40.35187	41.69829
Country	.0850017	.0880833	0.97	0.363	-.1181188	.2881222
_cons	.0839983	.1704374	0.49	0.635	-.309031	.4770277
Segment	-.0005913	.0242125	-0.02	0.981	-.0564254	.0552428
_cons	.2464666	.067471	3.65	0.006	.0908781	.4020551
B value	4.28e-06	.0002855	0.01	0.988	-.000654	.0006625
_cons	.2418725	.2179336	1.11	0.299	-.2606832	.7444282
Field strength	-.0388117	.034145	-1.14	0.289	-.1175501	.0399267
_cons	.3428749	.0878949	3.90	0.005	.1401889	.545561
Directions	-.0104812	.0119193	-0.88	0.405	-.0379672	.0170048
_cons	.2894789	.0534199	5.42	0.001	.1662924	.4126653

Note: Coef: Coefficient; Std. Err: Standard error; Conf. Interval: Confidence interval; _cons: Constant.

## Discussion

LDH is a common cause of lumbar and sacral nerve compression and has a high clinical incidence, severely affecting the physical and mental health of patients. Although conventional MRI can determine if a herniated disc is compressing the nerve root, it cannot determine the extent of damage and the intrinsic structure of the nerve root after compression. However, an electromyogram (EMG) can be used to examine the nerves in the compressed nerve root area; however, it can prove to be traumatic for the patient and psychological factors can partially influence the results [30]. Thus, DTI has been introduced for the assessment of nerve injury due to LDH. The FA and ADC values commonly employed in DTI can be applied to measure the degree of nerve damage caused by LDH and have been widely used in clinical practice recently [14,17,25,26]. Furthermore, a significant correlation between FA and ADC values and clinical symptoms has been reported. Studies also show that higher Japanese Orthopaedic Association (JOA) scores were associated with higher FA and lower ADC values, and lower Roland–Morris Disability Questionnaire (RDQ) scores were associated with higher FA and lower ADC values [31]. Moreover, pre-operative or post-operative symptom improvements were also accompanied by changes in DTI values [19,32]. These studies reveal that DTI is an essential tool for the assessment of neurological abnormalities due to LDH as it can not only pinpoint the location of the nerve root compression for clinical reference before surgery but also complement the severity of clinical symptoms to achieve a higher degree of disease assessment. Additionally, it can also assess postoperative nerve recovery without causing adverse effects. Thus, a meta-analysis of DTI on the compressed and healthy sides of patients with LDH is necessary to demonstrate the validity of fMRI techniques in the diagnosis of nerve injury caused by LDH.

To the best of our knowledge, this meta-analysis is the first to perform a quantitative diagnosis of nerve root compression due to LDH using DTI. While assessing neurological status, the representative indicators are FA values and ADC values [8]. Thus, we focused on the changes in these indicators on the compressed and healthy sides using DTI. The results revealed nerve damage to some extent after the compression of the nerve by a herniated disc, with the affected side showing a decrease in FA values and an increase in ADC values compared to the healthy side via DTI analysis. This indicates the potential of DTI in the clinical assessment of the degree of nerve root damage due to LDH. The sensitivity of DTI detection is influenced by many factors, including population type, compression segments, age, gender, countries, MRI parameters and others [3]. Hence, to reduce the bias of the results due to demographic characteristics, the healthy side of the same segment in the same patient was selected as a control in this study.

To further investigate the source of heterogeneity, subgroup analysis was performed based on the varying field strength. The results showed that for FA value, the I^2^ value of 1.5T increased to 96.2% while that of 3.0T decreased to 74.3%; for ADC value, the I^2^ value of 1.5T decreased to 67.2%, while that of 3.0T decreased to 70.1%. This suggests that different field strengths could lead to high heterogeneity of DTI measurement results. With the continuous development of imaging technology, 3.0T is gradually replacing 1.5T in clinical use owing to its higher definition. Nonetheless, 3.0T MRI is now mostly used for the diagnosis of nerve injury [33]. Nevertheless, our results showed that although the strength of the magnetic field leads to an increase in heterogeneity, the subgroup analysis reveals that the results obtained support the conclusions of this study, regardless of whether it is 1.5T or 3.0T. Additionally, there exist certain differences in the conventional parameter settings of DTI in the included literature, including b-value, directions, TE, TR, FOV, Matrix and other variables. Different parameter settings have varying effects on the results of neurological measurements; therefore, it is vital to set an appropriate and reasonable reference range.

Meta-regression analysis further revealed that segment and field strength did not have a significant effect on the results, regardless of the FA or ADC values. Contrastingly, in FA, there may be some effect of year of publication, country, b value and directions on the results, which may be related to the fact that there are more values of them and less literature included. However, the small size of included literature makes the meta-regression results less robust.

Various clinical studies have demonstrated a reduction in FA values on the damaged side of the nerve whereas the ADC values remain controversial [19,25,31,34,35]. For instance, Wu et al. [19] found no statistically significant difference in ADC values between the compressed lateral nerve and the contralateral nerve root at the same stage (P = 0.517). ADC values mainly respond to the ability of water molecules to diffuse in the tissues. When nerve compression is significant, various factors lead to nerve oedema, myelin lysis, axonal swelling and subsequently increased diffusion of water molecules inside and outside the nerve, ultimately leading to an increase in ADC values [16,19,27,35,36]. Furthermore, Zhang et al. [14] reported a moderate negative correlation between nerve root compression severity for FA (r = -0.646, P < 0.01) and a positive correlation for ADC (r = 0.408, P < 0.01). However, it has been speculated that ADC values might not change significantly when there is a lack of oedema, inflammatory lesions and demyelination of the nerve root [12]. The results of our meta-analysis show that when lumbar disc herniation causes nerve compression, the decrease in FA values of the nerve on the compressed side is clear and an increase in ADC values is observed, which can serve as a reference in clinical practice.

## Limitation

This study has certain limitations. First, the number of studies included was less and although certain conclusions were drawn, the results still lack robustness. Second, the heterogeneity of this article is high, and although subgroup analysis and heterogeneity tests were conducted, the main cause of heterogeneity remains unknown. Third, some of the extracted data were incomplete and some higher-quality documents were excluded because of the lack of primary data. Fourth, DTI detection is influenced by various factors. Although subgroup analysis analysed the magnetic field strength, the small sample size makes the results less reliable. Moreover, various interference factors in the parameters of DTI need to be unified and coordinated to better and more accurate results. Additionally, this article could not obtain a PROSPERO registration number in time; however, this overlook will be rectified soon. Furthermore, the samples included in the literature are small sample studies; therefore, further validation is required using a large sample and multicentric studies.

## Conclusions

This study demonstrated that DTI is essential for the non-destructive assessment of the operational status of nerves compressed by LDH. The nerve on the compressed side showed a significant decrease in FA values and an increase in ADC values compared to the healthy side via DTI. Thus, the diagnosis of nerve damage due to LDH using DTI is confirmed to be accurate. This mode of testing can be used as a reference in clinical practice.

## Supporting information

S1 Checklist (DOCX)

## References

[1] Wei FL, Zhou CP, Zhu KL, Du MR, Liu Y, Heng W, et al. Comparison of Different Operative Approaches for Lumbar Disc Herniation: A Network Meta-Analysis and Systematic Review. Pain Physician. 2021;24(4):E381-+. 34213864

[2] Yin MC, Mo W, Wu HY, Xu JH, Ye J, Chen N, et al. Efficacy of Caudal Epidural Steroid Injection with Targeted Indwelling Catheter and Manipulation in Managing Patients with Lumbar Disk Herniation and Radiculopathy: A Prospective, Randomized, Single-Blind Controlled Trial. World Neurosurgery. 2018;114:E29–E34. doi: 10.1016/j.wneu.2018.01.162 29410375

[3] Liang WS, Han B, Hai Y, Yin P, Chen YX, Zou CY. Diffusion tensor imaging with fiber tracking provides a valuable quantitative and clinical evaluation for compressed lumbosacral nerve roots: a systematic review and meta-analysis. European Spine Journal. 2021;30(4):818–28. doi: 10.1007/s00586-020-06556-8 32748258

[4] Urquhart DM, Zheng YL, Cheng AC, Rosenfeld JV, Chan P, Liew S, et al. Could low grade bacterial infection contribute to low back pain? A systematic review. Bmc Medicine. 2015;13. doi: 10.1186/s12916-015-0267-x 25609421 PMC4320560

[5] Amin RM, Andrade NS, Neuman BJ. Lumbar Disc Herniation. Current Reviews in Musculoskeletal Medicine. 2017;10(4):507–16. doi: 10.1007/s12178-017-9441-4 28980275 PMC5685963

[6] Brayda-Bruno M, Tibiletti M, Ito K, Fairbank J, Galbusera F, Zerbi A, et al. Advances in the diagnosis of degenerated lumbar discs and their possible clinical application. European Spine Journal. 2014;23:S315–S23. doi: 10.1007/s00586-013-2960-9 23978994

[7] Daoust J, Schaffer J, Zeighami Y, Dagher A, Garcia-Garcia I, Michaud A. White matter integrity differences in obesity: A meta-analysis of diffusion tensor imaging studies. Neuroscience and Biobehavioral Reviews. 2021;129:133–41. doi: 10.1016/j.neubiorev.2021.07.020 34284063

[8] Liu C, Li HW, Wang L, Zhu L, Jiang XF, Yang MJ, et al. Optimal parameters and location for diffusion tensor imaging in the diagnosis of carpal tunnel syndrome: a meta-analysis. Clinical Radiology. 2018;73(12). doi: 10.1016/j.crad.2018.08.015 30314809

[9] Zhu FZ, Zeng L, Gui S, Liu Y, Wang YL, Cao XJ, et al. The Role of Diffusion Tensor Imaging and Diffusion Tensor Tractography in the Assessment of Acute Traumatic Thoracolumbar Spinal Cord Injury. World Neurosurgery. 2021;150:E23–E30. doi: 10.1016/j.wneu.2021.01.146 33561552

[10] Wang WZ, Liu X, Yang ZY, Wang YZ, Lu HT. Diffusion tensor imaging of the hippocampus reflects the severity of hippocampal injury induced by global cerebral ischemia/reperfusion injury. Neural Regeneration Research. 2022;17(4):838–44. doi: 10.4103/1673-5374.322468 34472484 PMC8530111

[11] Andreisek G, White LM, Kassner A, Sussman MS. Evaluation of Diffusion Tensor Imaging and Fiber Tractography of the Median Nerve: Preliminary Results on Intrasubject Variability and Precision of Measurements. American Journal of Roentgenology. 2010;194(1):W65–W72. doi: 10.2214/AJR.09.2517 20028893

[12] Sakai T, Miyagi R, Yamabe E, Fujinaga Y, N Bhatia N, Yoshioka H. Diffusion-weighted imaging and diffusion tensor imaging of asymptomatic lumbar disc herniation. The journal of medical investigation: JMI. 2014;61(1–2):197–203. doi: 10.2152/jmi.61.197 . 24705766

[13] Shi Y, Zong M, Xu XQ, Zou YF, Feng Y, Liu W, et al. Diffusion tensor imaging with quantitative evaluation and fiber tractography of lumbar nerve roots in sciatica. European Journal of Radiology. 2015;84(4):690–5. doi: 10.1016/j.ejrad.2015.01.006 25631881

[14] Zhang JL, Zhang F, Xiao FX, Xiong ZG, Liu D, Hua T, et al. Quantitative Evaluation of the Compressed L5 and S1 Nerve Roots in Unilateral Lumbar Disc Herniation by Using Diffusion Tensor Imaging. Clinical Neuroradiology. 2018;28(4):529–37. doi: 10.1007/s00062-017-0621-9 28828579

[15] Chen YY, Lin XF, Zhang F, Zhang X, Hu HJ, Wang DY, et al. Diffusion Tensor Imaging of Symptomatic Nerve Roots in Patients with Cervical Disc Herniation. Academic Radiology. 2014;21(3):338–44. doi: 10.1016/j.acra.2013.11.005 24361075

[16] Li CT, Wang QZ, Xiao WF, Hui YY, Zhao B. 3.0T MRI tractography of lumbar nerve roots in disc herniation. Acta Radiologica. 2014;55(8):969–75. doi: 10.1177/0284185113508179 24132770

[17] Shi Y, Zou YF, Feng Y, Dou WQ, Ding HY, Wang CB, et al. A quantitative and clinical evaluation of nerve roots in lumbosacral radiculopathy using diffusion tensor imaging. Japanese Journal of Radiology. 2020;38(3):222–30. doi: 10.1007/s11604-019-00913-4 31865529

[18] Wu WF, Liang J, Chen Y, Chen AH, Wu B, Yang Z. Microstructural changes in compressed nerve roots treated by percutaneous transforaminal endoscopic discectomy in patients with lumbar disc herniation. Medicine. 2016;95(40). doi: 10.1097/MD.0000000000005106 27749591 PMC5059094

[19] Wu WF, Liang J, Ru N, Zhou CS, Chen JF, Wu YD, et al. Microstructural Changes in Compressed Nerve Roots Are Consistent With Clinical Symptoms and Symptom Duration in Patients With Lumbar Disc Herniation. Spine. 2016;41(11):E661–E6. doi: 10.1097/BRS.0000000000001354 26656057

[20] Moher D, Liberati A, Tetzlaff J, Altman DG, Grp P. Preferred Reporting Items for Systematic Reviews and Meta-Analyses: The PRISMA Statement. Annals of Internal Medicine. 2009;151(4):264–W64. doi: 10.7326/0003-4819-151-4-200908180-00135 19622511

[21] Stang A. Critical evaluation of the Newcastle-Ottawa scale for the assessment of the quality of nonrandomized studies in meta-analyses. European Journal of Epidemiology. 2010;25(9):603–5. doi: 10.1007/s10654-010-9491-z 20652370

[22] Zeng L, Du Y, Liao H, Xie M, Zhang Y, Huang R, et al. EFFICACY OF 3.0 T MR DIFFUSION TENSOR IMAGING IN DIAGNOSING NERVE ROOT INJURY IN UNILATERAL ACUTE LUMBAR DISC HERNIATION. Chinese Journal of Pain Medicine. 2018;24(10):748–52. CSCD:6352282.

[23] Chen J, Lin H, Chen K. Application of Magnetic Resonance Diffusion Tensor Imaging in Sacral Nerve Root Lesions. Journal of Clinical Radiology. 2020;39(3):533–6. CSCD:6725260.

[24] Cui T, Li S. Correlation between DTI features of compressed nerve roots and clinical symptoms in patients with lumbar disc herniation. Chinese Journal of Medical Imaging Technology. 2017;33(12):1869–73. CSCD:6129392.

[25] He A, Wang WZ, Qiao PF, Qiao GY, Cheng H, Feng PY. Quantitative Evaluation of Compressed L4-5 and S1 Nerve Roots of Lumbar Disc Herniation Patients by Diffusion Tensor Imaging and Fiber Tractography. World Neurosurgery. 2018;115:E45–E52. doi: 10.1016/j.wneu.2018.03.134 29597019

[26] Shi Y, Zhao F, Dou WQ, Ding HY, Zou YF, Feng Y, et al. Quantitative Evaluation of Intraspinal Lumbar Disc Herniation-related Lumbosacral Radiculopathy Before and After Percutaneous Transforaminal Endoscopic Discectomy Using Diffusion Tensor Imaging. Spine. 2021;46(13):E734–E42. doi: 10.1097/BRS.0000000000003925 33399366

[27] Balbi V, Budzik JF, Duhamel A, Bera-Louville A, Le Thuc V, Cotten A. Tractography of lumbar nerve roots: initial results. European Radiology. 2011;21(6):1153–9. doi: 10.1007/s00330-010-2049-3 21240648

[28] Li JQ, Cui H, Liu ZP, Sun YP, Zhang F, Sun YC, et al. Utility of diffusion tensor imaging for guiding the treatment of lumbar disc herniation by percutaneous transforaminal endoscopic discectomy. Scientific Reports. 2019;9. doi: 10.1038/s41598-019-55064-3 31822704 PMC6904469

[29] Liu Z, Sun Y, Cui H, Cui J, Zhang W. Feasibility of diffusion tensor imaging quantitative analysis in evaluation of nerve repair after minimally invasive surgery for lumbar disc herniation. Chinese Journal of Radiology. 2020;54(4):356–9. CSCD:6689766.

[30] Li YZ, Zhang XY, Dai J, Wang JS, Wu H, Liu JH, et al. Changes in the Flexion-Relaxation Response After Percutaneous Endoscopic Lumbar Discectomy in Patients with Disc Herniation. World Neurosurgery. 2019;125:E1042–E9. doi: 10.1016/j.wneu.2019.01.238 30776518

[31] Eguchi Y, Oikawa Y, Suzuki M, Orita S, Yamauchi K, Suzuki M, et al. Diffusion tensor imaging of radiculopathy in patients with lumbar disc herniation PRELIMINARY RESULTS. Bone & Joint Journal. 2016;98B(3):387–94. doi: 10.1302/0301-620X.98B3.36036 26920965

[32] Wu WF, Liang J, Chen Y, Chen AH, Wu YD, Yang Z. Microstructural changes are coincident with the improvement of clinical symptoms in surgically treated compressed nerve roots. Scientific Reports. 2017;7. doi: 10.1038/srep44678 28294192 PMC5353690

[33] Shan W, Wang XL. Clinical application value of 3.0T MR diffusion tensor imaging in grade diagnosis of gliomas. Oncology Letters. 2017;14(2):2009–14. doi: 10.3892/ol.2017.6378 28781644 PMC5530196

[34] Belykh E, Kalinin AA, Patel AA, Miller EJ, Bohl MA, Stepanov IA, et al. Apparent diffusion coefficient maps in the assessment of surgical patients with lumbar spine degeneration. Plos One. 2017;12(8). doi: 10.1371/journal.pone.0183697 28846710 PMC5573303

[35] Zhao BF, Yang FX, Guan L, Li XB, Hu YM, Zhang CL, et al. Fast Independent Component Analysis Algorithm-Based Diagnosis of L5 Nerve Root Compression and Changes of Brain Functional Areas Using 3D Functional Magnetic Resonance Imaging. Journal of Healthcare Engineering. 2021;2021. doi: 10.1155/2021/5063021 34336154 PMC8321732

[36] Tournier JD, Mori S, Leemans A. Diffusion Tensor Imaging and Beyond. Magnetic Resonance in Medicine. 2011;65(6):1532–56. doi: 10.1002/mrm.22924 21469191 PMC3366862

